# Complete Genome Sequence of *Streptococcus salivarius* HSISS4, a Human Commensal Bacterium Highly Prevalent in the Digestive Tract

**DOI:** 10.1128/genomeA.01637-15

**Published:** 2016-02-04

**Authors:** Johann Mignolet, Laetitia Fontaine, Michiel Kleerebezem, Pascal Hols

**Affiliations:** a Institut des Sciences de La Vie, Université catholique de Louvain, Louvain-la-Neuve, Belgium; b Host Microbe Interactomics Group, Wageningen University, Wageningen, The Netherlands

## Abstract

The human commensal bacterium *Streptococcus salivarius* plays a major role in the equilibrium of microbial communities of the digestive tract. Here, we report the first complete genome sequence of a *Streptococcus salivarius* strain isolated from the small intestine, namely, HSISS4. Its circular chromosome comprises 1,903 coding sequences and 2,100,988 nucleotides.

## GENOME ANNOUNCEMENT

*Streptococcus salivarius* is a Gram-positive bacterium and a member of the salivarius group of streptococci that preferentially colonizes the upper part of the human digestive tract. Remarkably, this commensal bacterium is one of the pioneer colonizers of oral and small intestine mucosal surfaces in newborns (1). It remains predominant in the oropharyngeal (1, 2) and gastrointestinal (3) tract throughout the human life span and is proposed to contribute to oral and gut health (4, 5). It was reported that *S. salivarius* might have specific inhibitory effects on pathogenic streptococci (6–8), or on bacteria involved in periodontal infections (9). In addition, this species is capable of *in vivo* modulating the periodontal and colon pathogen-induced inflammatory response (10, 11). Based on these beneficial effects, it is marketed as a probiotic (12). However, the impact of indigenous *S. salivarius*, as well as nonnative strain supplementation on gut microbiota, remains poorly investigated.

The *Streptococcus salivarius* HSISS4 was originally isolated in an ileostomy effluent sample from a 79-year-old male (13, 14) but was also suggested to colonize the oral cavity since closely related isolates were identified from saliva samples (15).

A draft genome of strain HSISS4 was previously assembled (13) using a combination of 454 and Illumina HiSeq sequencing technologies, which yielded a chromosome fragmented into 150 contigs (GenBank accession no. ASKD01000000). Here, we carried out a *de novo* hybrid assembly from a PacBio sequencing (generation of 151,277 sequence reads with an average length of about 3,800 bp) that ensures approximately 144-fold sequence coverage of the entire genome and an Illumina HiSeq2500 sequencing (generation of approximately 1,250,000 high-quality paired-end 125 bp-sequence reads) that enables gap closure and corrections of PacBio sequencing errors.

We performed a systematic reannotation of the whole genome, linking the previous gene annotation to our new gene nomenclature. The protein-coding sequences (CDSs), tRNAs, and rRNAs were predicted using the Rapid Annotation using Subsystem Technologies server (16). Moreover, we performed a proteomic comparative analysis with the *Streptococcus thermophilus* LMG18311 strain (17) in order to enrich our genomic database. In parallel, the metabolic pathways were reconstructed by using the Kyoto Encyclopedia of Genes and Genomes (18).

The circular chromosome of *S. salivarius* HSISS4 comprises 2,100,988 bp with an overall G+C content of 40.2%. It encodes 1,903 putative CDSs, among which 1,533 (81.6%) encode proteins with a predicted biological function. The genome encompasses 33 repeated elements and 68 tRNA genes and 18 rRNA genes allocated in 6 operons.

Comparative analyses with *S. thermophilus* LMG18311 and the oral isolate *S. salivarius* JIM8777 revealed that their genome encodes orthologs of HSISS4 genes for 1,378 and 1,695 CDSs, respectively (cutoff: 80% protein identity and 80% length coverage). This may indicate that, within the *salivarius* cluster, species and strains have specifically evolved to better cope with their ecological niche.

### Nucleotide sequence accession number.

The complete genome sequence of *Streptococcus salivarius* strain HSISS4 has been deposited at GenBank under accession number CP013216.

## References

[1] Seow WK, Lam JH, Tsang AK, Holcombe T, Bird PS 2009 Oral *Streptococcus* species in pre-term and full-term children—a longitudinal study. Int J Paediatr Dent 19:406–411. doi:10.1111/j.1365-263X.2009.01003.x. 19732193

[2] Bik EM, Long CD, Armitage GC, Loomer P, Emerson J, Mongodin EF, Nelson KE, Gill SR, Fraser-Liggett CM, Relman DA 2010 Bacterial diversity in the oral cavity of 10 healthy individuals. ISME J 4:962–974. doi:10.1038/ismej.2010.30. 20336157 PMC2941673

[3] Wang M, Ahrné S, Jeppsson B, Molin G 2005 Comparison of bacterial diversity along the human intestinal tract by direct cloning and sequencing of 16S rRNA genes. FEMS Microbiol Ecol 54:219–231. doi:10.1016/j.femsec.2005.03.012. 16332321

[4] Burton JP, Wescombe PA, Cadieux PA, Tagg JR 2011 Beneficial microbes for the oral cavity: time to harness the oral streptococci? Benef Microbes 2:93–101. doi:10.3920/BM2011.0002. 21840808

[5] Teughels W, Kinder Haake S, Sliepen I, Pauwels M, Van Eldere J, Cassiman JJ, Quirynen M 2007 Bacteria interfere with *A. actinomycetemcomitans* colonization. J Dent Res 86:611–617. doi:10.1177/154405910708600706. 17586706

[6] Ogawa A, Furukawa S, Fujita S, Mitobe J, Kawarai T, Narisawa N, Sekizuka T, Kuroda M, Ochiai K, Ogihara H, Kosono S, Yoneda S, Watanabe H, Morinaga Y, Uematsu H, Senpuku H 2011 Inhibition of *Streptococcus mutans* biofilm formation by *Streptococcus salivarius* FruA. Appl Environ Microbiol 77:1572–1580. doi:10.1128/AEM.02066-10. 21239559 PMC3067281

[7] Tamura S, Yonezawa H, Motegi M, Nakao R, Yoneda S, Watanabe H, Yamazaki T, Senpuku H 2009 Inhibiting effects of *Streptococcus salivarius* on competence-stimulating peptide-dependent biofilm formation by *Streptococcus mutans*. Oral Microbiol Immunol 24:152–161. doi:10.1111/j.1399-302X.2008.00489.x. 19239643

[8] Chatterjee C, Paul M, Xie L, van der Donk WA 2005 Biosynthesis and mode of action of lantibiotics. Chem Rev 105:633–684. doi:10.1021/cr030105v. 15700960

[9] Lévesque C, Lamothe J, Frenette M 2003 Coaggregation of *Streptococcus salivarius* with periodontopathogens: evidence for involvement of fimbriae in the interaction with *Prevotella intermedia*. Oral Microbiol Immunol 18:333–337. doi:10.1034/j.1399-302X.2003.00085.x. 12930529

[10] Sliepen I, Van Damme J, Van Essche M, Loozen G, Quirynen M, Teughels W 2009 Microbial interactions influence inflammatory host cell responses. J Dent Res 88:1026–1030. doi:10.1177/0022034509347296. 19828891

[11] Kaci G, Goudercourt D, Dennin V, Pot B, Doré J, Ehrlich SD, Renault P, Blottière HM, Daniel C, Delorme C 2014 Anti-inflammatory properties of *Streptococcus salivarius*, a commensal bacterium of the oral cavity and digestive tract. Appl Environ Microbiol 80:928–934. doi:10.1128/AEM.03133-13. 24271166 PMC3911234

[12] Wescombe PA, Heng NC, Burton JP, Chilcott CN, Tagg JR 2009 Streptococcal bacteriocins and the case for *Streptococcus salivarius* as model oral probiotics. Future Microbiol 4:819–835. doi:10.2217/fmb.09.61. 19722837

[13] Van den Bogert B, Boekhorst J, Herrmann R, Smid EJ, Zoetendal EG, Kleerebezem M 2013 Comparative genomics analysis of *Streptococcus* isolates from the human small intestine reveals their adaptation to a highly dynamic ecosystem. PLoS One 8:e83418. doi:10.1371/journal.pone.0083418. 24386196 PMC3875467

[14] Van den Bogert B, Meijerink M, Zoetendal EG, Wells JM, Kleerebezem M 2014 Immunomodulatory properties of *Streptococcus* and *Veillonella* isolates from the human small intestine microbiota. PLoS One 9:e114277. doi:10.1371/journal.pone.0114277. 25479553 PMC4257559

[15] Van den Bogert B, Erkus O, Boekhorst J, de Goffau M, Smid EJ, Zoetendal EG, Kleerebezem M 2013 Diversity of human small intestinal *Streptococcus* and *Veillonella* populations. FEMS Microbiol Ecol 85:376–388. doi:10.1111/1574-6941.12127. 23614882

[16] Aziz RK, Bartels D, Best AA, DeJongh M, Disz T, Edwards RA, Formsma K, Gerdes S, Glass EM, Kubal M, Meyer F, Olsen GJ, Olson R, Osterman AL, Overbeek RA, McNeil LK, Paarmann D, Paczian T, Parrello B, Pusch GD, Reich C, Stevens R, Vassieva O, Vonstein V, Wilke A, Zagnitko O 2008 The RAST server: Rapid Annotations using Subsystems Technology. BMC Genomics 9:75. doi:10.1186/1471-2164-9-75. 18261238 PMC2265698

[17] Bolotin A, Quinquis B, Renault P, Sorokin A, Ehrlich SD, Kulakauskas S, Lapidus A, Goltsman E, Mazur M, Pusch GD, Fonstein M, Overbeek R, Kyprides N, Purnelle B, Prozzi D, Ngui K, Masuy D, Hancy F, Burteau S, Boutry M, Delcour J, Goffeau A, Hols P 2004 Complete sequence and comparative genome analysis of the dairy bacterium *Streptococcus thermophilus*. Nat Biotechnol 22:1554–1558. doi:10.1038/nbt1034. 15543133 PMC7416660

[18] Ogata H, Goto S, Sato K, Fujibuchi W, Bono H, Kanehisa M 1999 KEGG: Kyoto Encyclopedia of Genes and Genomes. Nucleic Acids Res 27:29–34. doi:10.1093/nar/27.1.29. 9847135 PMC148090

